# Enhancing automated right‐sided early‐stage breast cancer treatments via deep learning model adaptation without additional training

**DOI:** 10.1002/mp.17682

**Published:** 2025-02-18

**Authors:** Michele Zeverino, Silvia Fabiano, Wendy Jeanneret‐Sozzi, Jean Bourhis, Francois Bochud, Raphaël Moeckli

**Affiliations:** ^1^ Institute of Radiation Physics Lausanne University Hospital and University of Lausanne Lausanne Switzerland; ^2^ Radiation Oncology Department Zurich University Hospital and University of Zurich Zurich Switzerland; ^3^ Radiation Oncology Department Lausanne University Hospital and University of Lausanne Lausanne Switzerland

**Keywords:** breast cancer, deep learning auto‐planning, model adaptation

## Abstract

**Background:**

Input data curation and model training are essential, but time‐consuming steps in building a deep‐learning (DL) auto‐planning model, ensuring high‐quality data and optimized performance. Ideally, one would prefer a DL model that exhibits the same high‐quality performance as a trained model without the necessity of undergoing such time‐consuming processes. That goal can be achieved by providing models that have been trained on a given dataset and are capable of being fine‐tuned for other ones, requiring no additional training.

**Purpose:**

To streamline the process for producing an automated right‐sided breast (RSB) treatment planning technique adapting a DL model originally trained on left‐sided breast (LSB) patients via treatment planning system (TPS) specific tools only, thereby eliminating the need for additional training.

**Methods:**

The adaptation process involved the production of a predicted dose (PD) for the RSB by swapping from left‐to‐right the symmetric structures in association with the tuning of the initial LSB model settings for each of the two steps that follow the dose prediction: the predict settings for the post‐processing of the PD (ppPD) and the mimic settings for the dose mimicking, respectively. Thirty patients were involved in the adaptation process: Ten manual plans were chosen as ground truth for tuning the LSB model settings, and the adapted RSB model was validated against 20 manual plans. During model tuning, PD, ppPD, and mimicked dose (MD) were iteratively compared to the manual dose according to the new RSB model settings configurations. For RSB model validation, only MD was involved in the planning comparison. Subsequently, the model was applied to 10 clinical patients. Manual and automated plans were compared using a site‐specific list of dose‐volume requirements.

**Results:**

PD for the RSB model required substantial corrections as it differed significantly from manual doses in terms of mean dose to the heart (+11.1 Gy) and right lung (+4.4 Gy), and maximum dose to the left lung (+6.4 Gy) and right coronary (+11.5 Gy). Such discrepancies were first addressed by producing a ppPD always superior to the manual dose by changing or introducing new predict settings. Second, the mimic settings were also reformulated to ensure a MD not inferior to the manual dose. The final adapted version of the RSB model settings, for which MD was found to be not significantly different than the manual dose except for a better right lung sparing (‐1.1 Gy average dose), was retained for the model validation. In RSB model validation, a few significant—yet not clinically relevant—differences were noted, with the right lung being more spared in auto‐plans (‐0.6 Gy average dose) and the maximum dose to the left lung being lower in the manual plans (‐0.8 Gy). The clinical plans returned dose distributions not significantly different than the validation plans.

**Conclusion:**

The proposed technique adapts a DL model initially trained for LSB cancer for right‐sided patients. It involves swapping the dose predictions from left to right and adjusting model settings, without the need for additional training. This technique—specific to a TPS—could be transposed to other TPS platforms.

## 1 INTRODUCTION

The progress of automation in radiation therapy treatment planning is swiftly progressing toward the aim of diminishing inter‐ and intra‐planner discrepancies, reducing planning time, and enhancing the standardization of the treatment planning process.^1^, ^2^, ^3^ Various methodologies have been employed to facilitate automated planning, with the most prevalent ones including wish‐list‐based iterative planning,^4^ knowledge‐based planning (KBP), and more sophisticated deep learning (DL) techniques.

KBP endeavors to predict the dose‐volume histograms (DVHs) of a candidate patient based on existing knowledge contained in the clinical plan database, utilizing pre‐defined features such as organ‐at‐risk (OAR) and target overlap.^5^, ^6^, ^7^ Conversely, DL‐based models avoid user‐predefined rules for dose prediction and, once adequately trained, are able to predict the three‐dimensional dose distribution for any given patient geometry.^8^, ^9^, ^10^, ^11^, ^12^ However, the efficacy of predicted dose (PD) distributions depends upon the quality of training data and the model training process to circumvent the “garbage‐in, garbage‐out” phenomenon.^13^

Data curation and model training remain the most time‐consuming processes in building a novel automated planning model capable of consistently generating outcomes adaptable to different patients' geometries.^14^ Data curation plays a pivotal role in determining the quantity and quality of the training data. A dataset that is too small may not reflect the heterogeneity of a patient population^13^ and at the same time an unrevised large dataset alone does not guarantee a good quality dataset. For instance, for an auto‐planning model, data curation is essential in discriminating the nature of plan outliers: if originating from sub‐optimal plans (dosimetric outliers), corrective measures are necessary through replanning; if related to patient anatomy (geometric outliers), replanning may not prove useful, and they can be incorporated into the model training.^15^ Therefore, large amounts of time are necessary to collect useful training data. Similarly, DL techniques take a long time to train a model due to the large number of trainable parameters; these can easily be millions for DL‐based auto‐planning models.^16^ Moreover, mitigating the overfitting problem during model training is essential to enhance the model's generalizability beyond the validation range,^15^ but that requires time as well as the use of any other data augmentation strategy to alleviate the effects of class imbalance. It is worth noting that in the specific case of dose prediction, data augmentation might also lead to the violation of the physical constraints when data transformations do not take into account the dependency of the dose distribution with the physics of the photon‐matter interaction.^17^, ^18^

To expedite the development of new models, several authors have explored strategies to minimize or eliminate the necessity for extensive model training. These include the development of multi‐purpose automated planning approaches with a limited number of training patients,^19^ the integration of manual clinical priorities to regulate models’ outputs trained with a small number of plans,^20^, ^21^ and the adaptation of existing models to different treatment techniques and patient orientations for the same treatment site.^22^, ^23^, ^24^

To the best of our knowledge, there have been no attempts thus far to adapt validated models for other treatment sites without requiring additional model training. Initially, this adaptation would require adjusting the PD, regardless of its nature, to align with distinct clinical requirements. The current commercial implementation of DL‐models in treatment planning systems (TPS) may prove to be efficient for this specific task.

In brief, the automated workflow consists of three sequential steps. First, the dose value per voxel is predicted using a trained 3D U‐NET convolution neural network (CNN).^25^ Subsequently, post‐processing is applied to alter the predicted DVH to potentially enhance dose quality, and post‐processed PD (ppPD) is resampled to reflect the DVH adjustments. Finally, the dose mimicking serves as the optimization algorithm to generate a clinical dose that is as closely aligned as possible to the post‐processed dose.

Both the PD post‐processing and the dose mimicking rely on a pre‐defined set of optimization functions, known as the model settings, that comes along with the trained auto‐planning model. These model settings can be customized for each optimization structure involved in dose prediction, providing flexibility for users to modify, remove, or introduce new settings. Such alterations influence the resulting clinical dose starting from the same initial PD, broadening the model's generalizability range. For example, adjustments to the model settings can prioritise the sparing of a specific OAR by modifying the related optimization functions in both post‐processing and dose mimicking settings.

This unique feature of DL‐based models facilitates further adaptation, particularly in scenarios like breast cancer treatment planning. These treatments typically exhibit near‐symmetrical characteristics concerning dose distribution in the left‐right direction on the axial plane. Given a one‐sided model, utilizing a technique capable of mirroring the PD and swapping the symmetric OARs model settings would suffice to initiate the model adaptation for the other side. Sub‐optimal dose predictions, as well as key anatomical differences that affect planning dose, including the position of the heart (shifted more toward the left) and the liver (entirely on the right side), can be addressed by tuning the original model settings. Consequently, it is theoretically feasible to adapt an existing left‐sided breast (LSB) auto‐planning model to the right side through adjustments in post‐processing and dose mimicking model settings, reducing the efforts in data curation and eliminating the need for an additional model training.

This study aims to demonstrate how a previously validated DL auto‐planning model developed for LSB under deep‐inspiration‐breath‐hold (DIBH) conditions can be adapted and utilized to generate auto‐plans for right‐sided breast (RSB) patients treated under free breathing conditions.

## 2 MATERIALS AND METHODS

The existing LSB auto‐planning model was adapted, validated, and clinically used for RSB patients. The model adaptation and validation involved selecting 30 previously treated RSB patients whose manual dose plans served as the ground truth. Ten of these patients were chosen for model adaptation, while the remaining 20 were used to validate the adapted model. Finally, the adapted model was employed to generate clinical plans, which were delivered to an additional 10 RSB patients.

### 2.1 Generating the auto‐planning process for adapting the existing DL model

**FIGURE 1:**
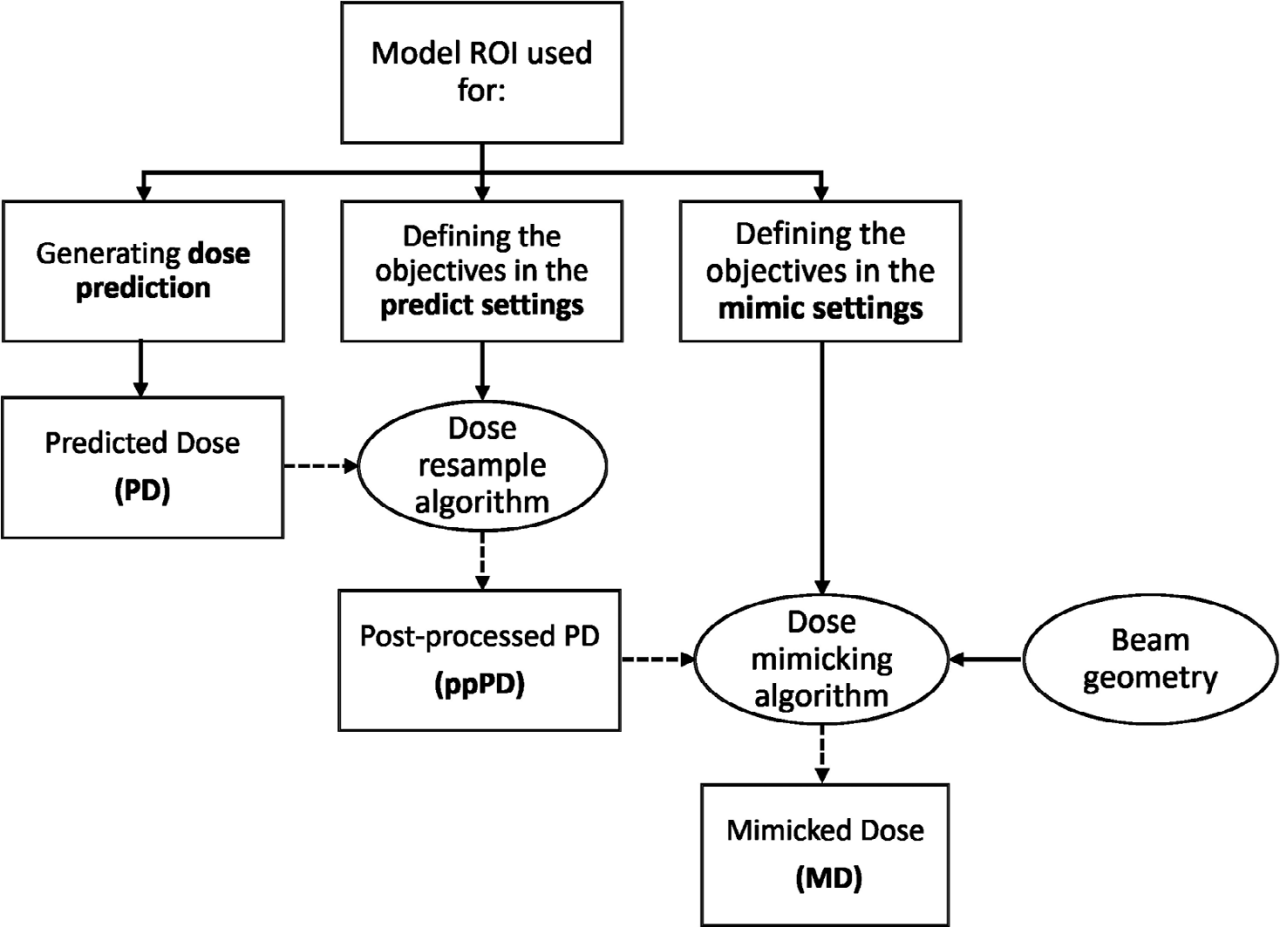
Structure of the auto‐planning module implemented in RayStation TPS. The Model ROI are used to generate the predicted dose (PD) and subsequently used with associated predefined and task‐specific objectives to formulate both the predict and mimic settings. Such settings are then used to produce the post‐processed predicted dose (ppPD) and the mimicked dose (MD) by means of their respective algorithms. Of note, the beam geometry is taken into account by the dose mimicking algorithm only to carry out the clinically deliverable MD. The dotted arrows show the automated workflow in its current TPS implementation.

The auto‐planning implementation in RayStation (RS) TPS [v12A, RaySearch Laboratories (RSL), Stockholm, Sweden] has been extensively detailed in previous studies.^12^, ^26^, ^27^ In summary, it operates according to regions‐of‐interest (ROIs) utilized as binary masks for model training, categorized as Model ROIs and defined separately for targets and OARs. For a new plan, the process initiates with the association of patient ROIs with the Model ROIs, followed by the manual configuration of beam geometry and irradiation technique. Subsequently, a fully automated optimization is initiated, where the three‐dimensional PD is inferred based on the patient's ROIs and is then post‐processed (ppPD) before undergoing a mimicking process that also serves as sequencing algorithm finding machine‐related deliverable beam segments to produce the mimicked dose (MD) that best match the ppPD.^28^ Therefore, the MD represents the only clinically deliverable dose at the end of the workflow. Each auto‐planning model has a task‐specific pre‐defined set of objectives that are tuned during model training and assigned to Model ROIs for use in both post‐processing and dose mimicking, named as predict and mimic settings, respectively (see Figure 1 for details).

Both the PD and ppPD are independent from the beam configuration. The PD is the dose output generated by the trained 3D U‐NET CNN for the given patient geometry. The post‐processing algorithm, as implemented in RS, consists of altering the DVHs of the PD using specific predict settings that will be introduced later in the paragraph. The PD is then resampled to fit the new DVHs by means of histogram matching, then producing the ppPD used as input for the mimicking algorithm. This intermediate step usually serves to ensure an input for the mimicking algorithm that is more clinically preferable than the U‐NET output, typically improving the target coverage or conformality and/or OARs dose sparing. The MD indeed depends on the beam geometry since the mimicking algorithm^29^ also optimizes the shape and fluence of each segment to reproduce the ppPD according to the model settings.

The model settings comprise task‐specific combinations of objectives, functions, and weights assigned to each Model ROI that differ in their formulation between predict and mimic settings. In the first case, they are built to ensure improvement of the dose distribution, while in the second case, they work to reproduce the same input (i.e., the ppPD) as closely as possible, being part of the voxel‐wise least squared error (LSE) minimization problem solved by the mimicking algorithm.^26^

The structure of the predict settings is composed of a dose‐volume objective associated with a function without the use of weighting factors, where the dose‐volume objective is structured as a goal type associated to a dose or dose‐volume threshold value.

The mimic settings have a similar structure making use of the same functions and dose‐volume objectives with the difference that they are alternative weighted objectives (i.e., the dose‐volume objective does not need to be associated to a function) applied to the ppPD during the mimicking process. This optimization process produces the clinical dose over a predefined number of iterations distributed in various cycles, allowing for the assignment of weights that can vary in each cycle for the same function or dose‐volume objective. According to the objective used, the weights can be applied to control the dose or the relative isodose values (isoweights) in a given ROI.

The mimic settings also comprise the “classic” objective functions available in RS that can be introduced at the beginning of a given cycles with a specific weight that can be modifed over the cycles as well.

For the formulation of the new predict settings, all three goal types available were used: *MinDose* (sets a minimum dose goal for the ROI), *MaxDose* (sets a maximum dose goal for the ROI), and *ReduceDose* (reduces the dose in a specified ROI). Among the various functions available, we used the *Aggressive* (sets the DVH values to at most or at least the specified dose value, depending on the goal type), *DVHShift* (shifts the DVH to a specified dose‐volume value), and *Reduction* (valid only for *ReduceDose*, it varies between 0 and 1, roughly 0%–100% fraction dose reduction in the given ROI). In this context, the value represents the dose goal, the dose‐volume goal or the dose reduction fraction according to the function used, respectively.

In the adaptation of the mimic settings, only the *MaxDose* was used both as function and dose objective with increasing weights and dose values per iteration cycle, along with the *MaxRefDose* function, aiming to reproduce the shape of various ppPD relative isodoses with different priorities also defined by increasing weights.

The complete list of predict and mimic settings is available in the user guide “RayStation 12A—Scripting Environments for Machine Learning.”^30^

In collaboration with RSL, we recently developed two DL models able to predict the dose distribution for simultaneous‐integrated‐boost (SIB) left‐sided early‐stage breast cancer treatments under DIBH. Both models were trained with 80 plans of patients referred to our institute and whose clinical dose distributions underwent a careful data curation process before being used for model training.^27^ One of these two models (named RSL‐Breast‐L‐4800‐SIB in RS v12A) was used to adapt the auto‐planning process from LSB to RSB as follows:

1. Run the existing RSL‐Breast‐L‐4800‐SIB DL model with a modified Model ROI association that involved the left‐to‐right swapping of both the planning target volumes PTV_Boost and PTV_Breast, and the symmetric OARs, while keeping the original Model ROI association for non‐symmetric OARs as presented in Table 1 to produce the PD. By employing this swapping technique, any alteration made to the original model settings was automatically applied to the corresponding symmetric organ, if applicable. This is because the model settings are associated with the Model ROI, regardless of their specific pairing. For example, adjusting a model setting for the left lung would consequently impact the right lung.
2. Evaluate the PD against the manual dose distribution. Define new predict settings to achieve a ppPD superior to the manual dose in terms of quality—that is with less dose to OARs and improved target coverage and homogeneity.
3. Run the dose mimicking of the ppPD by copying the beam configuration from manual plans. Evaluate the MD against both the ppPD and manual dose. Define new mimic settings to minimize the MD deviation from the ppPD and simultaneously ensure a MD quality not inferior to the manual plans.

**TABLE 1:** The Model ROI association defined to produce the right‐sided breast predicted dose.

Model ROI	Associated patient ROI
PTV_High	*PTV_Boost*
PTV_Low	*PTV_Breast*
PTV_Low—PTV_High	*PTV_Breast—PTV_Boost*
PTV_Low_crop_1 cm	*PTV_Breast—(PTV_Boost + 1 cm)*
Right breast	*Left Breast*
Heart	Heart
Left coronary	*Right Coronary*
Right coronary	*Left Coronary*
Right lung	*Left Lung*
Left lung	*Right Lung*
Liver	Liver
Spinal canal	Spinal canal

*Note*: ROI that were swapped from left to right with respect to the original model association are reported in italics. PTV_low_crop_1 cm was associated with the difference between the PTV_Breast and the PTV_Boost with 1 cm isotropic margin. The other structures are described in the text.

### 2.2 Treatment planning

Dose prescription was 48 Gy to the PTV_Boost and 42.4 Gy to the PTV_Breast, simultaneously delivered in 16 fractions and normalised to the median PTV_Boost volume. Plans were designed with OARs that were automatically generated using the DL contouring model available in RS and recently validated.^31^ Both PTVs were generated by expanding their respective manually contoured clinical target volumes (CTVs) by 5 mm and then cropping them 3 mm under the skin.

The simulation CT resolution was 1 × 1 × 2 mm^3^. A volumetric‐modulated‐arc‐therapy (VMAT) technique involving two reversed 6MV flattening‐filter‐free partial arcs was employed and delivered with a Synergy c‐arm linac equipped with Agility multi‐leaf collimator (MLC) (Elekta AB, Stockholm, Sweden). Initial and final gantry angles varied according to the patient geometry with an arc span ranging from 210° to 230°, the collimator angles were set to 5° and 355° per arc, respectively, the maximum delivery time per arc was set to 75 s, and the number of control points was set every 3° of gantry spacing. Finally, the dose calculation grid [collapsed cone convolution (CCC) algorithm] was set to 3 × 3 × 3 mm^3^.

Manual plans were optimized according to the list of dose‐volume constraints reported in Table 2. Auto‐plans were designed copying the same beam arrangement used for manual planning. Regardless of the planning approach, the dose optimization process consisted of 240 iterations, equally divided into four cycles. Each cycle produced a clinical dose at the end, which was then used as the starting solution for the subsequent cycle.

**TABLE 2:** Dose‐volume constraints and planning comparison for the patients involved in the model tuning.

			Median (Range)			
Structure	Figure of merit	Requirement	(a) Reference dose (RD)	(b) Predicted dose (PD)	(c) Post‐processed PD (ppPD)	(d) Mimicked Dose (MD)
PTV_Boost	Average dose	≤ 48 Gy	47.9^c^ (47.8−48.0)	47.8^c^ (47.8−47.9)	48.2^a,b^ (47.8−48.4)	47.9 (47.8−47.9)
	D98%	> 45.6 Gy	45.8 (44.8−46.4)	46.0 (45.2−46.4)	45.5^d^ (44.3−46.0)	46.1^c^ (45.3−46.3)
	D2%	< 49.4 Gy	49.1^b^ (48.6−49.7)	48.6^a,d^ (48.5−48.9)	48.8^d^ (48.5−48.8)	49.3^b,c^ (48.7−50.1)
	CI	> 0.6	0.64^c^ (0.47−.70)	0.70 (0.62−0.76)	0.88^a,d^ (0.77−0.9)	0.66^c^ (0.47−0.74)
PTV_Breast	D95%	> 40.3 Gy	40.6^c^ (39.8−41.1)	39.8^c,d^ (34.2−40.2)	42.3^a,b^ (41.5−42.3)	40.9^b^ (40.1−41.0)
	CI	> 0.8	0.91^b^ (0.83−0.95)	0.96^a,c,d^ (0.93−0.98)	0.90^b^ (0.84−0.93)	0.92^b^ (0.86−0.95)
PTV_Breast—PTV_Boost	V44.5 Gy	< 10 %	8.0^c^ (5.4−11.3)	5.5^d^ (2.7−6.7)	2.8^a,d^ (1.0−3.7)	10.7^b,c^ (5.3−16.2)
	V46.6 Gy	< 2 %	2.1^c^ (1.2−2.5)	1.4 (0.5−1.9)	0.7^a^ (0.4−2.2)	1.6 (0.7−2.0)
Left breast	Average dose	< 2 Gy	1.1^c^ (0.5−2.1)	0.7 (0.6−1.1)	0.5^a,d^ (0.5−0.8)	1.1^c^ (0.5−3.2)
	D1%	< 7 Gy	5.2^b,c^ (3.0−8.7)	2.4^a^ (2.0−3.7)	1.8^a,d^ (1.7−2.3)	5.3^c^ (2.6−13.3)
Heart	Average dose	< 1 Gy	0.8^b,c^ (0.7−1.2)	1.9^a,c,d^ (1.2−3.3)	0.2^a,b,d^ (0.1−0.4)	0.8^b,c^ (0.6−1.1)
	D1%	< 3 Gy	2.2^b^ (1.7−3.2)	13.4^a,c,d^ (9.5−20.6)	1.8^b^ (1.7−1.8)	2.3^b^ (1.6−3.4)
Left coronary	D1%	< 1 Gy	0.9^c^ (0.6−1.6)	2.0^c^ (0.9−2.4)	0.3^a,b,d^ (0−0.3)	0.9^c^ (0.6−1.4)
Right coronary	D1%	< 3 Gy	2.0^b^ (1.6−3.1)	13.5^,c,d^ (6.8−20.4)	1.8^b^ (1.7−1.8)	1.8^b^ (1.5−2.7)
Right lung	Average dose	< 6 Gy	6.1^c^ (4.5−8.2)	10.5^c,d^ (8.3−11.3)	3.8^a,b,d^ (2.5−4.0)	5.0^b,c^ (4.2−6.3)
	V5Gy	< 25 %	27.1^c^ (21.4−37.8)	51.7^c,d^ (41.0−61.0)	14.6^a,b^ (11.6−16.4)	22.1^b^ (19.0−28.7)
	V10Gy	< 15 %	17.5^c^ (12.5−26.8)	40.7^c,d^ (32.2−46.2)	11.8^a,b^ (9.3−13.3)	12.8^b^ (10.0−17.4)
	V20Gy	< 8 %	10.4^c^ (6.6−16.6)	19.5^c,d^ (14.5−23.2)	5.2^a,b^ (3.8−6.9)	7.4^b^ (5.1−10.7)
	V40Gy	< 1%	0.2^b^ (0−0.1)	0.1^a,d^ (0−0.1)	0.1 (0−0.1)	0.1^b^ (0−0.1)
Left lung	Average dose	< 1 Gy	0.5^c^ (0.4−1.2)	1.1^c^ (0.7−1.4)	0.1^a,b,d^ (0.1−0.2)	0.7^c^ (0.4−2.3)
	D1%	< 4 Gy	1.8^b^ (1.4−4.1)	8.2^a,c^ (3.8−14.0)	0.3^b,d^ (0.3−0.3)	3.0^c^ (1.3−8.9)
Liver	Average dose	< 2 Gy	0.8^c^ (0.4−2.5)	2.1^c^ (0.4−6.1)	0.3^a,b,d^ (0.1−5.8)	0.9^c^ (0.4−2.1)
Spinal canal	Maximum dose	< 3 Gy	1.0^b,c^ (0.6−2.5)	3.6^a^ (1.0−6.0)	3.5^a^ (1.0−5.8)	3.0 (1.0−5.9)
External—PTVs	V44.1 Gy	< 10 cm^3^	1.8^c^ (0.2−3.1)	0.6 (0−2.3)	0.3^a^ (0−1.2)	1.2 (0.2−3.8)

*Note*: Statistically significant differences (*p * <  0.05) between dose distributions shown with a, b, c, and d superscripts with a = Test versus Reference Dose (RD); b = Test versus Predicted Dose (PD); c = Test versus Post‐Processed PD (ppPD); d Test versus Mimicked Dose (MD).

### 2.3 Model tuning and validation

Thirty right‐sided early breast cancer patients who were manually planned were retrospectively selected and included in the study. We assessed the need for ethical and/or legal approval and concluded that no approval was required as the patients’ data were fully anonymized for the present study.

Ten patients were used to iteratively modify the model settings for both the ppPD and the MD (model tuning), while the remaining 20 were used to validate the auto‐planning process. As the quality of the dose distribution may depend on the breast volume and shape,^32^ the patients’ selection was done to maximize the breast volume as well as the breast concavity ranges in both tuning and validation groups. Given the full range of anatomic features for the 30 patients involved, they were divided into tuning and validation groups ensuring that each group had a similar range of anatomic variability as the initial sample. The PTV_Breast was used as a surrogate for the breast volume, while the breast concavity was measured as the maximum extent of the right lung beyond the breast tangent over all CT slices. Patients’ statistics and anatomic features are reported in Table S1 and Figure S1 of the Supplementary Material, respectively.

The criteria to move from the tuning to the validation phase was to bring all auto‐plans having each figure of merit reported in Table S2 of the Supplementary Material with a MD better or at least not statistically significantly worse than the reference dose (RD, i.e., the manual dose).

The normal distribution of variables was first checked with the Shapiro–Wilk test,^33^ and since not all figures of merit were normally distributed, the non‐parametric Kruskal–Wallis test^33^ was used to compare the median values of RD, PD, ppPD, and MD. The differences were considered statistically significant if *p* < 0.05 for all cases, and a post‐hoc pairwise comparison of medians was followed with Bonferroni correction to reduce the whole type I error for pairwise comparisons. Each auto‐plan was also analysed in terms of pass/fail dose‐volume requirements for all the figures of merit.

Furthermore, to assess the model's ability to generalize predictions for the non‐symmetric OARs that remained unchanged in the Model ROI association, we evaluated the influence of the distance between the PTVs, heart, and liver on the heart maximum dose and the liver mean dose, respectively. This was done by comparing the Pearson correlation coefficients of the linear fit between the PD and the RD across all tuning plans, based on the final configuration of the model settings. A two‐tailed Student's *t*‐test was used to assess statistical significance (*p* < 0.05). During model validation, plans were compared in terms of RD versus MD only. Similarly, a non‐parametric test (Wilcoxon signed rank) was used to assess significant differences (*p* < 0.05) between the median values of the manual and auto‐plans. In addition, the manual plans used for validation were compared against those used for tuning to check dose consistency between the two groups by means of the Wilcoxon rank sum test (significant difference if *p* < 0.05). Finally, the analysis of failed objectives occurrences with respect to the clinical requirement was analyzed for each plan.

### 2.4 Clinical implementation

The validated auto‐planning process was applied to design and deliver the plans of 10 new patients referred to our institute for early‐stage right breast cancer treatments. The auto‐plans were reviewed by an experienced radiation oncologist, who required additional post‐automated optimization if deemed necessary. No manual plans were used for comparison. Clinical and validation cohorts were compared in terms of dose distribution to assess statistically significant differences (*p* < 0.05) using once again the Wilcoxon rank‐sum test because of different sizes between samples. Furthermore, the achievement of the clinical goals requirements was investigated. Patient‐specific quality assurance (psQA) was performed for each clinical plan using the Octavius 2D array system (PTW, Freiburg, Germany) and evaluated with the gamma analysis (3%/3 mm criteria, global maximum dose normalization).

## 3 RESULTS

All data presented in the following text and tables refer to the median and range (minimum and maximum value) values calculated over the number of patients per cohort (tuning = 10, validation = 20, clinical = 10).

### 3.1 Model tuning

Manual plans generally met the clinical requirements, with the exception of a slight overdosage observed in the right lung. Specifically, the mean dose exceeded the limit by 0.1 Gy, and for V5, V10, and V20, the dose was 2% higher than the prescribed volume limits. Outliers that were considered clinically relevant with respect to the constraints' limits were found in two patient cases, showing low dose conformality for the PTV_Boost (CI: ‐0.13) and an increased average dose to the right lung (+2.2 Gy). The PD was aligned, although with some minor deviations with the manual dose for both PTVs, while it was significantly larger than manual dose and out of tolerance for the heart (+1.1  and +11.1 Gy, for the average and maximum dose, respectively), the left lung (+6.4 Gy for the average dose), and the spinal canal (+2.6 Gy for the maximum dose). It was also found out of tolerance for the right lung (+4.4 Gy for the average dose) and right coronary (+11.5 Gy for the maximum dose), although the differences compared to the manual dose were not significant. To address this suboptimal dose prediction, a list of new or modified (with respect to their original formulation) predict settings was iteratively adjusted to produce the ppPD. The comparison between new and old predict settings is reported in Table S3 of the Supplementary Material. In the following, the description of modified settings always refers to the settings associated with the Model ROI, which may affect the opposite and symmetric OAR (Associated ROI) according to the ROI mapping reported in Table 1.

To improve the PTVs coverage, the dose values of the *MinDose* and *MaxDose* objectives associated with the *Aggressive* function for the PTV_Low were slightly increased. Similarly, for the PTV_High, the *MinDose* was increased from 46  to 47 Gy, and the dose‐volume requirement (*DVHShift)* of 48 Gy from 75% to 80% of the PTV_Boost volume. For the Heart, the *ReduceDose* objective value was increased from 0.2 to 0.6, and two new *MaxDose* objectives associated with the *Aggressive* function were applied to the whole OAR geometry and the OAR region more distal from the PTVs. For the right lung (Model ROI), a new *MaxDose* objective was introduced to limit the dose spillage in the left lung (Associated ROI). For the left lung (Model ROI), the value of the existing *ReduceDose* objective was increased from 0.1 to 0.5 to reduce the dose overall in the right lung (Associated ROI). For the liver, a new *ReduceDose* objective was introduced with a value of 0.8, and finally, the value of *MaxDose* associated with the *Aggressive* function for both coronaries was lowered with respect to their original formulation.

The ppPD produced with such changes of predict settings was observed to be significantly better than the manual dose for almost all structures involved. In particular, the target coverage and conformity were improved, and all the PD values out of tolerance were re‐aligned to the clinical requirements successfully (see Table 2 for details).

Similarly, the mimic settings were adjusted iteratively to achieve or improve the manual dose, minimizing the ppPD deterioration. The list of the original mimic settings is too extended to be reported; therefore, only the settings that were adapted are listed in Table S4 of the Supplementary material.

For the left lung (Model ROI), the weights were increased for the original *MaxRefDose* function in order to maintain the quality of the ppPD. *MaxDose* values were reduced for the heart, the left lung (Model ROI), and the left coronary (Model ROI), while they were relaxed for the right breast (Model ROI). Finally, one new RS objective function was introduced, and two RS objectives were modified: a fake constraint for the PTV_High (minimum dose = 0 Gy) was applied to help the optimization algorithm solve the problem more rigorously and adhere to the ppPD as much as possible, and two maximum dose objectives were decreased for both coronaries. The use of constraints in RS significantly slows down the optimization process; therefore, the constraint for the PTV_High was used only in the last of the four iteration cycles to avoid a large increase of the optimization time.

**FIGURE 2:**
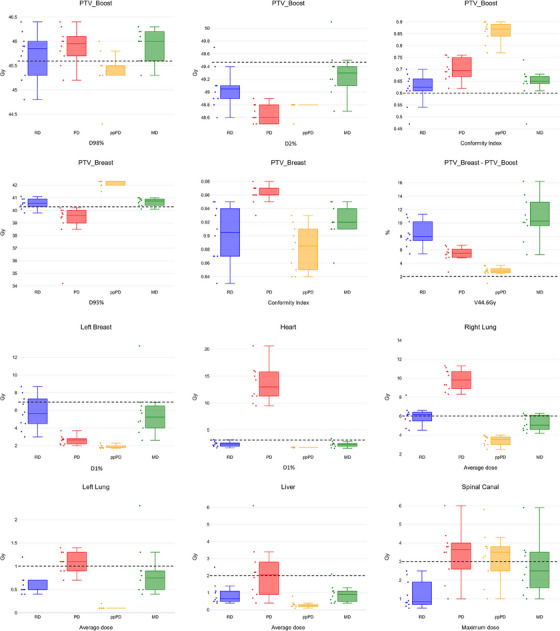
Box‐plots showing the differences in terms of reference dose (RD, i.e., the manual dose), predicted dose (PD), post‐processed PD (ppPD), and mimicked dose (MD) for the 10 tuning patients for the main dosimetric figure of merits for targets and OARs (upper and lower panel, respectively). The solid lines within the boxes represent the median value. The dashed lines represent the clinical requirement as reported in Table 2 per each figure of merit. This was reported per each subplot with the exception of the conformity index for the PTV_Breast that was found always above the threshold (CI > 0.8) that was lower than the minimum value observed.

As reported in Table 2, the MD median values generally met the clinical requirements, except for the average value of the PTV_Boost D2% and the PTV_Breast‐PTV_Boost V44.5 Gy, which slightly exceeded the limits by 0.2 Gy and 0.7%, respectively. There was no significant difference observed between RD and MD, with the latter being superior for right lung sparing, (‐1.1 Gy average dose), albeit at the cost of a small increase in the left lung and spinal canal maximum doses (+ 1.2  and +2 Gy on average, respectively). Clinically relevant outliers with respect to the constraints' limits were found in three cases: in one case, D2% for the PTV_Boost exceeded the limit by 1 Gy; in another, D1% for the left breast was significantly larger (13.3 Gy vs. 7 Gy), resulting in an average dose of 3.2 Gy (+1.2 Gy above the limit), and in the last case, D1 for the left lung was notably higher (8.9 Gy  vs. 4 Gy), resulting in an average dose of 2.3 Gy (+1.3 Gy above the limit). Finally, the MD values of V44.5 Gy for the PTV_Breast‐PTV_Boost were systematically higher than the RD (10.7% vs. 8%), as well as for the spinal canal maximum dose (3 Gy  vs. 1 Gy), although these differences were not statistically significant (see Figure 2 for details).

**FIGURE 3:**
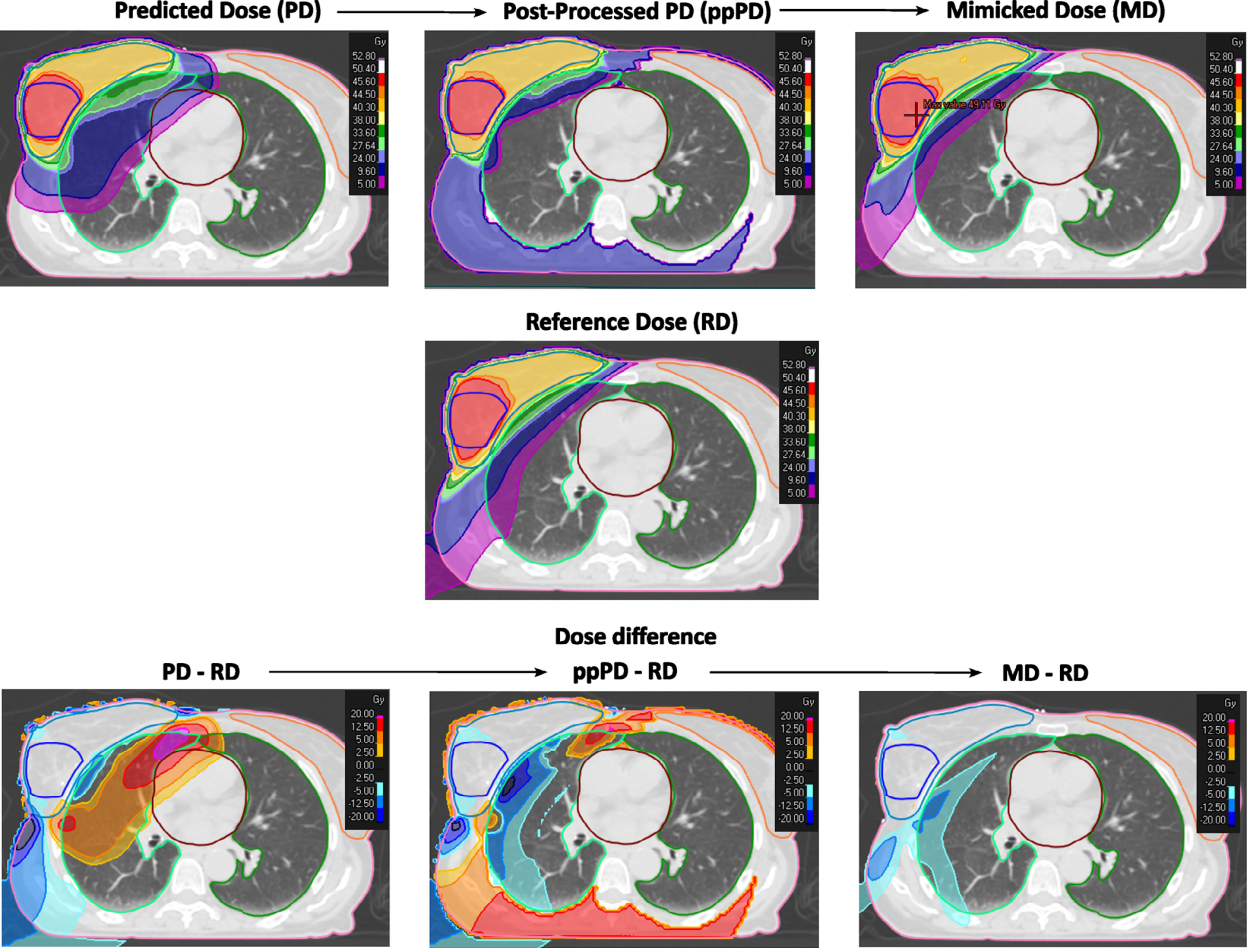
Sequential comparison between predicted dose, post‐processed predicted dose, and mimicked dose against the reference dose (RD) are shown in the upper panel. The lower panel also shows the corresponding dose difference versus RD.

An example of how the dose distribution was modified from PD to MD passing through the ppPD is shown in Figure 3 for a validation case. The low‐dose bath (approximately 30% of the prescription dose) visible in blue for the ppPD was introduced with a specific function assigned to the ROI = (external—all OARs) in the original predict settings to ensure more room for the choice of beam entry angles. As no mimic settings were used for the same ROI while it was the case for all other ROIs, such a low‐dose bath was automatically erased during the dose mimicking optimization process.

**FIGURE 4:**
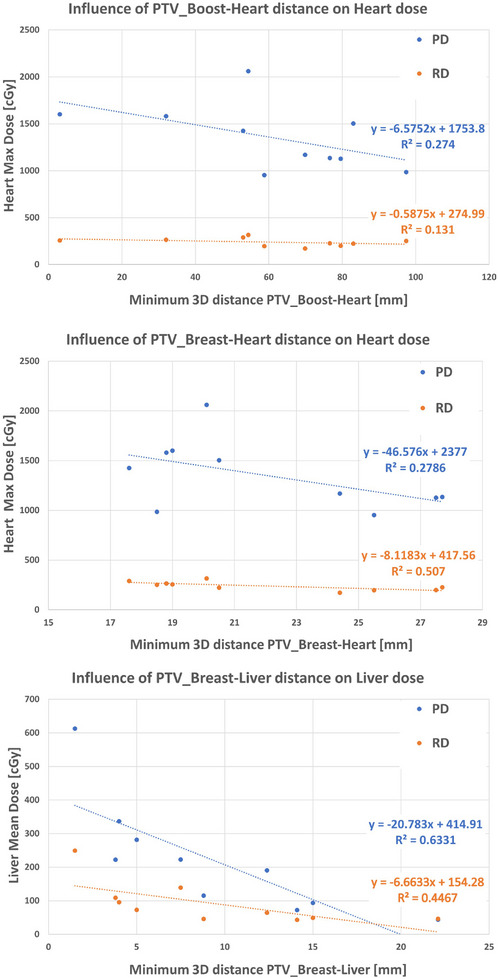
Comparison of the predicted dose (PD) and reference dose (RD) for the heart maximum dose and liver mean dose in the 10 tuning patients, plotted as a function of the minimum three‐dimensional distance between both PTVs and heart (upper panel: PTV_Boost, middle panel_ PTV_Breast), and PTV_Breast and liver (lower panel).

The influence of the distance between the PTVs and non‐symmetric OARs on the maximum dose to the heart and the mean dose to the liver is shown in Figure 4, where the linear fits of the PD and RD were compared. In general, RD was less influenced by the distances, which was expected, as the dose to the asymmetric OARs was controlled by adjusting the dose‐volume objectives during manual planning. On the other hand, the PD was more influenced by the distance, increasing as the distance decreased. The differences in the Pearson coefficients were found to be statistically significant for the heart maximum dose (PD = ‐0.53, *p* = 0.12 vs. RD = ‐0.71, *p* = 0.02) and for the liver mean dose (PD = 0.8, *p* < 0.01 vs. RD = ‐0.67, *p* = 0.03).

### 3.2 Model validation

No significant differences were observed for median values of clinical requirements of manual plans between tuning and validation patients, suggesting the consistency between the two groups in terms of plan quality as shown in Table S5 of Supplementary Material.

For the PTVs, auto‐plans were significantly (yet not clinically relevant) superior to manual plans in terms of dose conformity and homogeneity. For the OARs, auto‐plans returned better sparing of right lung (‐0.6 Gy in terms of average dose) at the cost of a small increase in the left lung D1 (+0.8 Gy). No other clinically relevant significant differences were observed. The full comparison between manual and auto‐plans is reported in Table 3.

**TABLE 3:** Reference dose (RD, i.e., manual dose) versus mimicked dose (MD, i.e., automated clinical dose) planning comparison for the validation patients.

			Median		Min		Max		
Structure	Figure of merit	Requirement	RD	MD	RD	MD	RD	MD	*p*
PTV_Boost	Average dose	≤ 48 Gy	47.9	47.9	47.8	47.8	48.0	48.0	0.29
	D98%	> 45.6 Gy	46.0	45.9	44.6	45.0	46.3	46.3	0.50
	D2%	< 49.4 Gy	49.1	49.2	48.6	48.9	49.6	50.0	0.04
	CI	> 0.6	0.62	0.64	0.40	0.49	0.74	0.78	0.05
PTV_Breast	D95%	> 40.3 Gy	40.6	40.6	40.1	40.2	41.1	41.1	0.84
	CI	> 0.8	0.88	0.93	0.79	0.86	0.94	0.96	<0.01
PTV_Breast—PTV_Boost	V44.5 Gy	< 10 %	7.1	8.3	4.1	4.8	13.7	14.5	0.31
	V46.6 Gy	< 2 %	1.9	1.2	0.9	0.9	4.8	2.5	<0.01
Left Breast	Average dose	< 2 Gy	0.8	1.2	0.4	0.5	2.4	2.1	0.22
	D1%	< 7 Gy	4.3	5.6	2.2	2.2	12.4	7.9	0.08
Heart	Average dose	< 1 Gy	0.8	0.8	0.4	0.5	1.2	1.0	0.7
	D1%	< 3 Gy	2.1	2.1	1.4	1.5	3.2	2.8	0.7
Left coronary	D1%	< 1 Gy	0.8	0.8	0.5	0.5	2.8	1.1	0.81
Right coronary	D1%	< 3 Gy	1.9	1.9	1.0	1.1	3.2	2.8	0.17
Right lung	Average dose	< 6 Gy	5.7	5.1	4.4	3.6	8.4	6.3	<0.01
	V5Gy	< 25 %	27.1	23.1	20.4	15.7	37.5	32.7	<0.01
	V10Gy	< 15 %	15.9	13.2	11.6	8.0	25.3	17.8	<0.01
	V20Gy	< 8 %	8.8	7.5	4.4	3.8	17.9	10.4	<0.01
	V40Gy	< 1%	0.1	0.1	0.0	0.0	0.8	0.7	0.43
Left lung	Average dose	< 1 Gy	0.5	0.6	0.3	0.5	1.1	1.2	0.02
	D1%	< 4 Gy	1.7	2.5	1.0	1.9	4.3	5.6	<0.01
Liver	Average dose	< 2 Gy	0.9	0.9	0.1	0.1	2.0	1.8	0.5
Spinal canal	Maximum dose	< 3 Gy	1.1	2.1	0.4	0.7	2.5	6.5	<0.01
External—PTVs	V44.1 Gy	< 10 cm^3^	2.3	1.1	0.1	0.2	7.0	2.7	<0.01

The overall numbers of failed dose‐volume objectives were 65 (13.5%) and 47 (9.7%) out of the total 480, for the manual and auto‐plans, respectively. As reported in Table 4, most of the occurrences that were clinically relevant were found for the manual plans, specifically for the left breast maximum dose (median deviation of +3.2 Gy from the objective) and the right lung mean dose (median deviation of +1.1 Gy from the objective). For auto‐plans, only in one case the left lung maximum dose was found to be larger than the objective (+1.6 Gy).

**TABLE 4:** A. Comparison of failed objectives between manual (RD) and automated (MD) planning approach for the 20 validation cases. B. Evaluation of the failed objectives for the 10 clinical cases was assessed for MD only.

			A. Validation cohort (*n* = 20)					B. Clinical cohort (*n* = 10)			
			Occurrences	Median deviation		Range deviation		Occurrences		Median deviation	Range deviation
Structure	Figure of merit	Requirement	RD	MD	RD	MD	RD	MD	MD		
PTV_Boost	Average dose	≤ 48 Gy	0	0	−	−	−	−	2	+ 0.1 Gy	(0.01, 0.2) Gy
	D98%	> 45.6 Gy	3	2	−0.1 Gy	−0.3 Gy	(−0.9, −0.01) Gy	(−0.6, −0.01) Gy	0	–	–
	D2%	< 49.4 Gy	2	3	0.1 Gy	0.1 Gy	(0.02, 0.2) Gy	(0.1, 0.5) Gy	3	+ 0.3 Gy	(0.2, 0.7) Gy
	CI	> 0.6	8	7	−0.05	−0.04	(−0.2, −0.01)	(−0.11, −0.01)	0	–	–
PTV_Breast	D95%	> 40.3 Gy	2	1	−0.2 Gy	−0.1 Gy	−	−	0	−	−
	CI	> 0.8	1	0	−0.01	−	–	–	1	−0.05	–
PTV_Breast—PTV_Boost	V44.5 Gy	< 10 %	4	7	1.4%	2.5%	(0.01, 3.7) %	(0.03, 4.5) %	5	3.7%	(0.2, 4.4) %
	V46.6 Gy	< 2 %	8	3	0.4%	0.2 %	(0.1, 2.8) %	(0.02, 0.5) %	4	0.5%	(0.2, 1.3) %
Left breast	Average dose	< 2 Gy	3	2	+0.3 Gy	+0.1 Gy	(0.2, 0.4) Gy	(0.01, 0.1) Gy	1	+ 0.1 Gy	–
	D1%	< 7 Gy	3	5	+3.2 Gy	+0.6 Gy	(2.5, 5.4) Gy	(0.2, 0.9) Gy	2	+ 0.8 Gy	(0.6, 1.1) Gy
Heart	Average dose	< 1 Gy	3	1	+0.2 Gy	+0.1 Gy	(0.1, 0.2) Gy	–	0	–	–
	D1%	< 3 Gy	1	0	+0.2 Gy	–	–	–	0	–	–
Left coronary	D1%	< 1 Gy	4	2	+0.4 Gy	+0.1 Gy	(0.05, 1.8) Gy	(0.01, 0.1) Gy	1	+ 0.2 Gy	–
Right coronary	D1%	< 3 Gy	2	0	0.2 Gy	−	(0.1, 0.2) Gy	−	0	–	–
Right lung	Average dose	< 6 Gy	6	4	+1.1 Gy	+0.2 Gy	(0.1, 2.4) Gy	(0.01, 0.3) Gy	1	+ 1.2 Gy	–
	V5Gy	< 25 %	5	3	3.7%	1.6%	(1.5, 7.5) %	(1.1, 2.7) %	2	4.4%	(0.6, 8.2) %
	V10Gy	< 15 %	5	0	5.4%	–	(0, 7.3) %	–	1	5.1%	–
	V20Gy	< 8 %	4	0	2.1%	–	(1.4, 5.9) %	–	4	0.6%	(0.1, 5.5) %
	V40Gy	< 1%	0	0	–	–	–	–	0	–	–
Left lung	Average dose	< 1 Gy	1	3	+0.1 Gy	+0.1 Gy	–	(0.01, 0.2) Gy	1	+ 0.2 Gy	–
	D1%	< 4 Gy	1	1	+0.3 Gy	+1.6 Gy	–	–	4	+ 0.3 Gy	(0.2, 2.6) Gy
Liver	Average dose	< 2 Gy	0	0	–	–	–	–	1	+ 0.2 Gy	–
Spinal canal	Maximum dose	< 3 Gy	0	3	–	+0.2 Gy	–	(0.2, 0.6) Gy	1	+ 0.6 Gy	–
External—PTVs	V44.1 Gy	< 10 cm^3^	0	0	–	–	–	–	0	–	–
			Total					Total			
			65	47					34		

### 3.3 Clinical implementation

No significant differences were observed between the clinical and validation cohorts, except for the V46.6 Gy for the PTV_Breast‐PTV_Boost that was larger in the clinical cohort than in the validation cohort (see Table 5 for the detailed comparison). Both such small discrepancies were due to the increase of the hotspots within the target observed for two clinical patients. Both patients exhibited a PTV_Breast volume smaller than the minimum value of the tuning range.

**TABLE 5:** Planning comparison between validation and clinical automated plans.

			Clinical plans (*n* = 10)		Validation plans (*n* = 20)		
Structure	Figure of merit	Requirement	Median	Range	Median	Range	*P*
PTV_Boost	Average dose	≤ 48 Gy	47.9	47.8−48.2	47.8	47.8−48.0	0.05
	D98%	> 45.6 Gy	45.9	45.8−46.1	45.9	45.0−46.3	0.81
	D2%	< 49.4 Gy	49.3	48.9−50.2	49.2	48.9−0.0	0.68
	CI	> 0.6	0.63	0.48−0.71	0.64	0.49−0.78	0.25
PTV_Breast	D95%	> 40.3 Gy	40.6	40.4−40.9	40.6	40.2−41.1	0.35
	CI	> 0.8	0.94	0.75−0.96	0.93	0.86−0.96	0.98
PTV_Breast—PTV_Boost	V44.5 Gy	< 10 %	10.3	6.6−14.4	8.3	4.8−14.5	0.2
	V46.6 Gy	< 2 %	1.9	1.2−3.3	1.2	0.9−2,5	0.01
Left breast	Average dose	< 2 Gy	1.4	0.9−2.1	1.2	0.5−2.1	0.25
	D1%	< 7 Gy	5.8	3.0−8.1	5.6	2.2−7.9	0.91
Heart	Average dose	< 1 Gy	0.9	0.7−1.0	0.8	0.5− 1.0	0.4
	D1%	< 3 Gy	2.2	1.8−2.7	2.1	1.5−2.8	0.88
A_Coronary_L	D1%	< 1 Gy	0.8	0.7−1.2	0.8	0.5−1.1	0.12
A_Coronary_R	D1%	< 3 Gy	1.8	1.6−2.4	1.9	1.1−2.8	0.95
Right lung	Average dose	< 6 Gy	4.4	3.7−7.2	5.1	3.6−6.3	0.09
	V5Gy	< 25 %	19.7	15.7−33.2	23.1	15.7−32.7	0.13
	V10Gy	< 15 %	10.9	6.6−20.1	13.2	8.0−17.8	0.15
	V20Gy	< 8 %	6.4	2.1−12.5	7.5	3.8−10.4	0.07
	V40Gy	< 1%	0.1	0−0.9	0.1	0−0.7	0.88
Left lung	Average dose	< 1 Gy	0.9	0.5 ‐ 1.2	0.6	0.5−1.2	0.33
	D1%	< 4 Gy	3.9	1.9−6.6	2.5	1.9−5.6	0.14
Liver	Average dose	< 2 Gy	0.9	0.2−2.2	0.9	0.1−1.8	0.62
Spinal canal	Maximum dose	< 3 Gy	2.7	0.7−4.0	2.1	0.7−6.5	0.59
External—PTVs	V44.1 Gy	< 10 cm3	1.5	0.2−7.7	1.1	0.2−2.7	0.5

The failed objectives were 34 out of the 240 total, that is, the 14% of the total as reported in Table 4. The deviations from the objectives were similar in magnitude to the ones observed in the validation group. Only in one case the outcome of the auto‐planning process was considered largely outside the clinical requirements because of the dose received by the right lung that exceeded the tolerance level of 1.2 Gy for the average dose. For this plan, a manual planning approach attempting to reduce the dose to the right lung to within the limit was not successful because it caused an increase of the left breast mean dose deemed clinically unacceptable. The median number of failed objectives per plan was 2.5 [range 0 (two cases) – 8 (one case)]

All psQA plans returned a gamma passing rate > 97% that was aligned with the usual results of manual planning psQA for early‐stage breast cancer treatments observed in our institute.

## 4 DISCUSSION

This study presented a successful attempt to develop an auto‐planning process for RSB treatments based on the adaptation of a pre‐configured DL model built and validated for left‐sided patients through the reconfiguration of the model settings used to generate the post‐processing of the PD and the dose mimicking. To our knowledge, it is the first time that an auto‐planning process is generated from a model for which the primary intent was not that of the final auto‐planning process, with the corollary that no additional model training was necessary.

Although training with sidedness can be used to address data limitations when cases are insufficient, the preferred approach is to train auto‐planning models without sidedness to ensure generalizability and enhance clinical applicability. Adapting a sided model, rather than training a new one from scratch, can help to reduce the time‐consuming data curation process, which becomes particularly challenging when both a large number of patients and complex plans are involved, while still aiming to maximize model performance and applicability.The method involved generated an unknown PD based on the swapping from left to right of the symmetric ROIs. This prediction, that was largely clinically unacceptable as expected, was then iteratively adjusted by modifying the model settings in each of the two sequential steps of the auto‐planning pipeline implemented in RS until a satisfactory outcome involving at least the same dose distribution quality of the manual plans was attained. As demonstrated, the procedure did not involve any model training but rather a careful selection of tuning patients as well as the reconfiguration of the model settings, and more specifically for the predict settings to generate the ppPD. Once finalised, the process was fully automated, requiring no different actions from the planner with respect to the use of the original model except for the left‐to‐right swapping of the symmetric structures during the model ROI association.

The full adaptation procedure required an initial understanding of the use of the functions associated with the model settings that was, however, demonstrably shorter than the time needed for data curation used for model training that, in our previous experience, took approximately 6 months all together (data curation + model training).^27^ Overall, the entire adaptation process took approximately 4 months, spanning from patient selection and familiarization with the model settings to model validation. The tuning phase, which was the most time‐consuming, lasted about 2 months.

The post‐processing tool available in RS was the key point for producing a dose that could be easily mimicked thereafter. In fact, most of the model settings that were modified were the predict settings, while the mimic settings were only slightly changed. This demonstrates how the latter were well conceived in their original formulation to guarantee an efficient mimicking optimization for the same treatment technique. It is worth noting here that an overly “ideal” ppPD would not have been mimicked correctly because the mimicking optimization algorithm also takes into account the beam segmentation, and so, the mimicking of an unrealistic ppPD would have diverged the optimization problem. A more careful revision of the mimic settings is then expected if this adapted workflow is adapted for a different treatment machine or technique. If, for instance, another kind of linac was used for the same VMAT technique, the mimic settings should have also taken into account the differences due to the beam energy, MLC characteristics, dose rate, etc.

The logic behind the new configuration of the predict settings was based on the mutual evaluation of the resulting PD after the Model ROI swapping and the original predict settings. To improve the dose distribution for both PTVs, increasing only the dose values of the associated existing functions was sufficient. On the other hand, for the OARs, it was necessary to introduce new settings that were not necessary in the original model for different reasons. Specifically, we had to introduce two new predict settings for the heart because in the original model the organ sparing was intrinsic among the training data whereas the new setting for the liver was needed since this OAR was outside the treated volume for LSB patients. In general, new prediction settings were necessary because the dose prediction outcomes using the swapping technique prioritized target coverage over patient anatomy. Specifically, for non‐symmetric OARs, the mean dose prediction for the heart and liver varied linearly with the organ‐to‐target distance, whereas the RD was less affected by this distance. As shown in Figure 4, the PD for the heart increased as the minimum distance between the heart and the targets decreased. Similarly, the PD for the liver increased with a reduction in its minimum distance from the PTV_Breast. However, this trend was not observed for the RD in the heart, and it was reduced in the liver. Figure S3 in the Supplementary Material also shows such differences in terms of both 3D dose distribution and DVH for a validation patient.

For clarity, a different Model‐ROI association, excluding the swapping technique, would have resulted in different PD outcomes for the OARs, requiring an alternative set of prediction settings compared to those used in this study. The difference in dose prediction between the swapped technique presented in this study and the unswapped one is shown in Figure S2 of the Supplementary Material for one of the tuning cases. The unswapped technique involved the original Model ROI association, with the exception of the contralateral breast. In this case, the swapping was necessary during the Model ROI association to prevent a conflict with the PTV_Breast (i.e., the right breast), which had to be associated with PTV_Low. The dose comparison demonstrated how the Model ROI association affected the PD, particularly for both lungs, while differences for the heart (non‐symmetric) and both PTVs remained negligible.

The key advantage of the swapping technique was its ability to retain the original structure of the model settings for any ROI, with only the settings parameters needing adjustment, thereby enhancing the model's adaptation efficiency.

The changes brought to the original mimic settings were based on the comparison between the post‐processed, mimicked, and manual dose. We found that a minor revision of the weights and dose values for the mimic settings was effective in reducing the dose deterioration typical of the dose mimicking optimization. In addition, a few “classic” RS objectives were introduced to ensure a MD not inferior to the manual dose. The main difference with respect to their original formulation was the introduction of the constraint for the PTV_boost (in the form of the RS objective function “Minimum dose = 0 Gy”) for the last cycle of iterations during the optimization process. The use of the constraints for the manual optimization in RS forces the optimization problem to be solved rigorously, avoiding the risk of getting a spike of the objective cost function value at the end of the optimization once all the mechanical and dosimetric limits are taken into account (i.e., final dose). The use of a constraint that, by definition, is always fulfilled by the optimization problem ensures that the constraint does not affect the optimization other than forcing it to be solved rigorously. We observed for some tuning patients that the use of the constraint avoided this issue, providing a final dose more similar to the one achieved in the last iteration of the optimization process.

It is worth noting that the method presented can be easily applied when adapting the DL‐based left‐sided model for other techniques used in breast cancer treatments such as IMRT, Hybrid VMAT, or TomoDirect (TD)^34^, ^35^ regardless of the treatment side, since the automated workflow in RS does not strictly require the use of the same treatment machine used for model training. In the case of machine adaption for the original left‐sided DL model, the VMAT‐based PD will need to be post‐processed to closely match the manually produced dose with the alternative technique. For machine and breast side adaptation, the Model ROI swapping technique needs to be used prior to post‐processing. In both cases, adapting the mimic settings may end up being more labour intensive than presented here because of the difference of the sequencing parameters intrinsic to each delivery technique. The final result of any adapted model does not rely on a unique strategy of adaptation, as different combinations of model settings may end up with similar results. We believe it is wise to recommend starting the adaptation process with small changes to the original model settings (preferably one setting at a time) and evaluate the impact of such changes on the planned dose to understand the correct use of the different functions available.

The revision of the model settings produced consistent results between the tuning and validation patients as well as when applied to new patients thus confirming a certain degree of auto‐planning process generalization.

Auto‐plans based on the adapted model were found to be better than manual plans in the validation phase. The PTV_Boost coverage and the CI for both targets were higher in auto‐plans, also the dose to the right lung was significantly less than in manual plans. For the remaining OARs, dose differences were not significant except for a small increase in dose for the contralateral lung (+0.1 Gy for the mean dose and +0.8 Gy for the maximum dose) and for the spinal canal (+1 Gy for the maximum dose) that were judged not clinically relevant. The higher dose observed in auto‐plans for the spinal canal was attributed to the low‐dose bath introduced in the ppPD to accommodate beam geometry variability. This could potentially have been mitigated by introducing a classical RS objective, such as controlling the maximum dose to the spinal canal, among the mimic settings. The auto‐planning process demonstrated its robustness for the clinical patients, for whom no significant differences were observed between validation and clinical plans.

Achieving high plan quality and broadening the validation range should also be the two main objectives when existing auto‐planning models undergo any type of adaptation. Wu et al. adapted through MLC resequencing a KBP model trained with VMAT plans to produce IMRT‐based plans for rectal cancer, obtaining better results than manual IMRT plans regardless of the patient orientation.^22^ Many studies^36^, ^37^, ^38^, ^39^ have also been conducted to extend existing KBP models to other treatment devices through the extraction of DVH constraints from new prediction models (trained with the dose distribution of the different machine) to be used to generate a machine‐specific optimization template able to reproduce the predicted DVH. This approach was also effective in generating high quality auto‐plans when the KBP model was trained with a small (< 50) number of plans.^37^ Due to the diversity of the methods employed in the design of the adapted workflow, it is difficult to directly compare between studies. However, similar to the previously mentioned studies, our auto‐plans performed better than the manual plans. In addition, we also provided an initial follow‐up of the performance of the model by applying it clinically to 10 patients. We observed a substantial homogeneity in terms of plan quality, except for a small yet significant increase of hot spots in the PTV Breast likely due to the presence of smaller PTV Breast volumes than used in the tuning and validation phases. Among clinical plans, in one case, the dose to the right lung was larger than expected. This was basically due to an unfavourable patient's geometry in particular for the shape of the PTV Breast that induced a large exposure of the right lung to high doses and its proximity to the left breast. We attempted to reduce the right lung dose via a manual planning approach, but this significantly increased the right breast dose and was deemed clinically unacceptable considering the age of the patient and the increased risk of developing a radiation induced cancer to the ipsilateral breast.^40^ Therefore, the auto‐plan was finally delivered.

The small number of patients used for the model tuning and validation may represent the main limitation of the study, and essentially when considering the distribution of the PTV Breast volume observed among these patients. In fact, despite the efforts made in broadening the range of breast volume, only a few patients had volumes larger than 1500 cm^3^, considered the minimal threshold for a large breast volume.^41^ Consequently, the performance of the auto‐planning method remains to be tested for patients who exhibit a large body mass index (BMI) or who are elderly, as both factors are correlated with large breast volumes.^42^ Our future work will focus on evaluating the adapted model for patients with anatomical features outside the validation range and potentially adjusting the model settings to further enhance its generalization ability.

Although the approach presented here can also be extended for other treatment sites featuring a similar left‐to‐right anatomical symmetry (for instance, locally advanced breast or unilateral head & neck cancers) once the one‐sided DL model is available, its applicability is limited to the RS environment only.

The sequential approach consisting of the dose prediction, the post‐processing of the PD, and the mimicking the ppPD was deemed necessary because the DL model (the 3D U‐NET) was trained using 3D dose distributions in conjunction with patient anatomy, only. This method, based on the work of McIntosh et al.,^8^ differs from other DL‐based auto‐plan generation approaches that employ more advanced model training techniques. For instance, some methods use Monte Carlo dose engines to account for continuous gantry and leaf motions during VMAT dose delivery.^43^ Others project fluence maps into the dose domain to train models for fixed‐beam IMRT plans^44^ or use fluence maps derived from KBP plans as inputs for plan generation in a commercial TPS environment.^45^ Another approach encodes linac machine parameters into the underlying anatomy to predict dose distributions for individual MLC shapes.^46^ These alternative training techniques have the advantage of incorporating plans or delivery characteristics directly into the training process, thereby eliminating the need for a separate optimization algorithm.

Nonetheless, despite the sequential method being closely related to the TPS used in this study, we believe that the general concept of side dose swapping can be extended to other commercial TPS that use different workflows to generate automated treatment plans starting from dose‐volume objective templates or dose predictions (either 3D dose or DVH). The recent implementation of auto‐planning in Pinnacle3 TPS (Philips Healthcare, Fitchburg, Wisconsin, USA) involves a template‐based optimization tool that iteratively adjusts optimization parameters to meet the predefined goals typical of each clinical protocol for both PTV and OARs.^47^, ^48^, ^49^ A one‐sided clinical protocol might initially be used for the contralateral side by changing the laterality of the symmetric structure to evaluate the preliminary results against a manual plan. Dose differences might then be reduced by iteratively tuning the objectives in the original protocol until the resulting automated dose matches or outperforms the manual dose, as we did during the tuning of the ppPD. In contrast to other approaches, the RapidPlan solution implemented in the Eclipse TPS (Varian Medical System, Palo Alto, California, USA) employs KBP techniques to predict the DVHs for OARs using principal component analysis (PCA) and regression analysis (RA).^50^, ^51^ While a model developed using both left‐sided and right‐sided data can accurately predict outcomes for either side, applying a model specifically trained for LSB cancer to right‐sided cases (or vice versa) may lead to inaccurate dose predictions for non‐symmetric OARs such as the heart and liver due to their differing geometric relationships with the PTV. To address this challenge, PCA and RA for these specific OARs should be recomputed for non‐symmetric structures and then integrated back into the original model. This will allow for an assessment of the model's ability to generalize to the contralateral side effectively.

Finally this paper would open new perspectives in terms of adapting existing models with new configurations of model settings, whichever their formulation, that do not require any symmetry. These configurations might potentially be used for specific clinical indications that do not largely diverge from the original training data.

## 5 CONCLUSION

The adaptation of an original DL‐model initially trained for left‐sided early breast cancer auto‐planning proved successful in its clinical implementation for right‐sided patients. This adaptation was achieved solely through the adjustment of model settings, eliminating the need for additional model training. Notably, both the post‐processed and the mimicked dose distributions were redesigned, resulting in improved outcomes compared to manual plans. While this technique is tailored to a specific TPS, it represents a pioneering strategy that could be transposed to other TPS platforms.

## CONFLICT OF INTEREST STATEMENT

The authors declare no conflicts of interest.

## Supporting information



Supporting information
