# Supplementary material for: Enhancing automated right‐sided early‐stage breast cancer treatments via deep learning model adaptation without additional training
Source: Med Phys. 2025 Feb 18;52(5):3280–97. doi: 10.1002/mp.17682 (PMC12059510; doi:10.1002/mp.17682)
Supplement: Supplementary file 1 — Supporting information [file MP-52-3280-s001.doc]

Supplementary file

|  |  | Tuning | Validation | Clinical |
| --- | --- | --- | --- | --- |
| Number of patients |  | 10 | 20 | 10 |
| Age (years) | Median | 66 | 70.5 | 58 |
|  | Min | 62 | 63 | 48 |
|  | Max | 78 | 79 | 77 |
|  |  |  |  |  |
| PTV_Breast (cm^3^) | Median | 796 | 792 | 813 |
|  | Min | 507 | 364 | 286 |
|  | Max | 1659 | 2218 | 1087 |

Table S1. Patients’ statistics listed in terms of age and PTV_Breast volume per cohort.


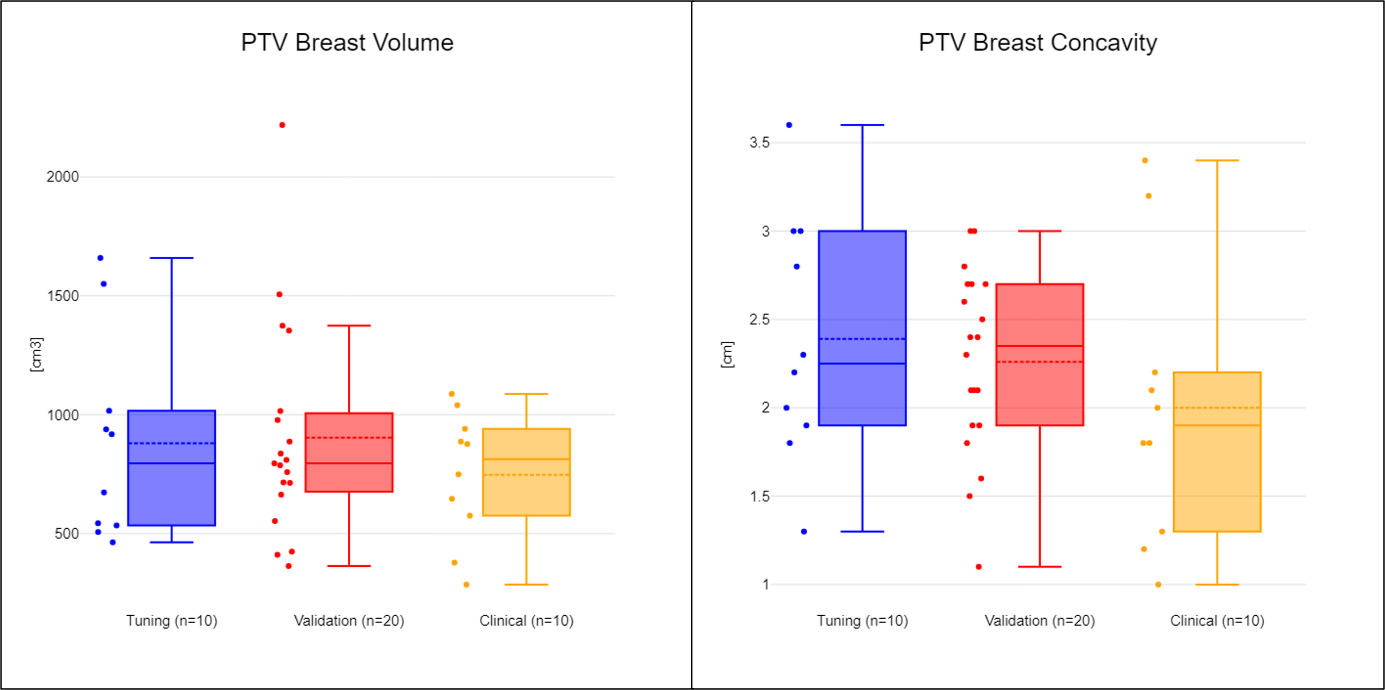


Figure S1. Box-plots comparing the PTV_Breast volume(left panel) and concavity (right panel) between the three cohorts of patients used in the study. The PTV Breast concavity was defined as the extent in cm of the right lung beyond the breast tangent measured in the breast midplane CT slice. No significant differences (p>0.05) were observed between the three cohorts for both the anatomic features. Solid and dashed lines within the boxes represent the median and average values, respectively.

| Structure | Figure of merit | Requirement |
| --- | --- | --- |
|  |  |  |
| PTV_Boost | Average dose | ≤ 48 Gy |
|  | D98% | > 45.6 Gy |
|  | D2% | < 49.4 Gy |
|  | CI | > 0.6 |
| PTV_Breast | D95% | > 40.3 Gy |
|  | CI | > 0.8 |
| PTV_Breast - PTV_Boost | V44.5Gy | < 10 % |
|  | V46.6Gy | < 2 % |
| Left Breast | Average dose | < 2 Gy |
|  | D1% | < 7 Gy |
| Heart | Average dose | < 1 Gy |
|  | D1% | < 3 Gy |
| Left Coronary | D1% | < 1 Gy |
| Right Coronary | D1% | < 3 Gy |
| Right Lung | Average dose | < 6 Gy |
|  | V5Gy | < 25 % |
|  | V10Gy | < 15 % |
|  | V20Gy | < 8 % |
|  | V40Gy | < 1% |
| Left Lung | Average dose | < 1 Gy |
|  | D1% | < 4 Gy |
| Liver | Average dose | < 2 Gy |
| Spinal Canal | Maximum dose | < 3 Gy |
| External - PTVs | V44.1Gy | < 10 cm3 |

Table S2. Dose-volume objectives used in the optimization of the manual plans as well as for automated vs manual planning comparison. External – PTVs refers to the external patient’s contour without both the PTV_Boost and PTV_Breast volumes.

| Predict Settings | | | | |
| --- | --- | --- | --- | --- |
| **Model ROI** | **Goal Type** | **Function** | **Adapted Model** | **Original Model** |
|  |  |  | **Value** | **Value** |
| *Target Type* | | |  |  |
| PTV_High | Min Dose | Aggressive | 47 Gy | 46 Gy |
| PTV_High - 5mm | Min Dose | DVH Shift | 48 Gy (Volume Threshold 0.8) | 48 Gy (Volume Threshold 0.75) |
| PTV_Low | Min Dose | Aggressive | 41.3 Gy | 40.3 Gy |
| PTV_Low - PTV_High | Max Dose | Aggressive | 46.2 Gy | 45.8 Gy |
| PTV_Low - (PTV_High+10mm) | Max Dose | Aggressive | 44.5 Gy | 44.1 Gy |
| PTV_Low - (PTV_High+5mm) | Max Dose | Aggressive | 45.5 Gy | 45 Gy |
|  |  |  |  |  |
| *OARs Type* | | |  |  |
| Heart | Reduce Dose | Reduction | 0.6 | 0.2 |
| Heart | Max Dose | Aggressive | 2 Gy | not present |
| Heart - (PTV_Low+40mm) | Max Dose | Aggressive | 1 Gy | not present |
| Left Lung | Reduce Dose | Reduction | 0.5 | 0.1 |
| Right Lung | Max Dose | Aggressive | 0.3 Gy | not present |
| Left Coronary + 5 mm | Max Dose | Aggressive | 2 Gy | 2.5 Gy |
| Right Coronary | Max Dose | Aggressive | 0.1 Gy | 0.8 Gy |
| Liver-PTV_Low | Reduce Dose | Reduction | 0.8 | not present |

Table S3. Predict settings comparison between the original and adapted models. They were also applied to some Boolean combinations of PTVs and OARs volumes. Four new settings were introduced for the heart, right lung, and liver.

| Mimic Settings | | | | | |
| --- | --- | --- | --- | --- | --- |
| **Functions** | | | | | |
| Model ROI | Function | Model | Weight | IsoDose | IsoWeights |
| Left Lung - PTV_Low | Max Ref Dose | Adapted | 0.15 | 78, 16.8, 8.3, 0 | 0.5, 1, 8, 12 |
|  |  | Original | 0.1 | 78, 16.8, 8.3, 0 | 0.5, 1, 4, 6 |
| Left Lung | Max Ref Dose | Adapted | 0.25 | 78, 16.8, 8.3, 0 | 0.5, 1, 4, 12 |
|  |  | Original | 0.2 | 78, 16.8, 8.3, 0 | 0.5, 1, 4, 6 |
|  |  |  |  |  |  |
| **ROI Goals** | | | | | |
| Model ROI | GoalType |  | Weights | Weight Scale per Run | Values (Gy) |
| Heart | Max Dose | Adapted | 2,2,2 | 0, 0.5, 1, 0 | 0.5, 0.75, 1 |
|  |  | Original | 2,2,2 | 0, 0.5, 1, 0 | 1, 3, 4.5 |
| Right Breast | Max Dose | Adapted | 2,2,2 | 0, 0.5, 1, 0 | 1.5, 3 |
|  |  | Original | 2,2,2 | 0, 0.5, 1, 0 | 1, 2 |
| Right Lung | Max Dose | Adapted | 1,1 | 0, 0.5, 1, 0 | 0.5, 1 |
|  |  | Original | 1,1 | 0, 0.5, 1, 0 | 1,2 |
| Left Coronary + 5mm | Max Dose | Adapted | 10,20 | 0,1,2,4 | 0.5, 0.75 |
|  |  | Original | 10,20 | 0,1,2,4 | 2.5, 3.5 |
|  |  |  |  |  |  |
| **RayStation Objective Functions** | | | | | |
| Model ROI | Function |  | Weight | Dose Level (Gy) | Constraint |
| PTV_High | Min Dose | Adapted | 1 | 0 | Yes |
|  |  | Original | Not present | | |
| ROI | Function |  | Weights | Dose Level (Gy) | Volume (cm3) |
| Left Coronary | Max DVH | Adapted | 3,6 | 1,2 | 0.01, 0.01 |
|  |  | Original | 3,6 | 2,3 | 0.01, 0.01 |
| Right Coronary | Max DVH | Adapted | 3 | 0.5 | 0.01 |
|  |  | Original | 3 | 1.5 | 0.01 |

Table S4. Mimic settings comparison between adapted and original models.

| Structure | Figure of merit | Requirement | Median | |  |
| --- | --- | --- | --- | --- | --- |
|  |  |  | Tuning (n=10) | Validation (n=20) | p-value |
| PTV_Boost | Average dose | ≤ 48 Gy | 47.9 | 47.9 | 0.47 |
|  | D98% | > 45.6 Gy | 45.8 | 46.0 | 0.31 |
|  | D2% | < 49.4 Gy | 49.1 | 49.1 | 0.91 |
|  | CI | > 0.6 | 0.64 | 0.62 | 0.52 |
| PTV_Breast | D95% | > 40.3 Gy | 40.6 | 40.6 | 0.31 |
|  | CI | > 0.8 | 0.91 | 0.88 | 0.26 |
| PTV_Breast - PTV_Boost | V44.5Gy | < 10 % | 8.0 | 7.1 | 0.32 |
|  | V46.6Gy | < 2 % | 2.1 | 1.9 | 0.58 |
| Left Breast | Average dose | < 2 Gy | 1.1 | 0.8 | 0.25 |
|  | D1% | < 7 Gy | 5.2 | 4.3 | 0.14 |
| Heart | Average dose | < 1 Gy | 0.8 | 0.8 | 0.23 |
|  | D1% | < 3 Gy | 2.2 | 2.1 | 0.31 |
| Left Coronary | D1% | < 1 Gy | 0.9 | 0.8 | 0.23 |
| Right Coronary | D1% | < 3 Gy | 2.0 | 1.9 | 0.27 |
| Right Lung | Average dose | < 6 Gy | 6.1 | 5.7 | 0.55 |
|  | V5Gy | < 25 % | 27.1 | 27.1 | 0.71 |
|  | V10Gy | < 15 % | 17.5 | 15.9 | 0.55 |
|  | V20Gy | < 8 % | 10.4 | 8.8 | 0.5 |
|  | V40Gy | < 1% | 0.2 | 0.1 | 0.81 |
| Left Lung | Average dose | < 1 Gy | 0.5 | 0.5 | 0.39 |
|  | D1% | < 4 Gy | 1.8 | 1.7 | 0.16 |
| Liver | Average dose | < 2 Gy | 0.8 | 0.9 | 0.56 |
| Spinal Canal | Max dose | < 3 Gy | 1.0 | 1.1 | 0.88 |
| External - PTVs | V44.1Gy | < 10 cm3 | 1.8 | 2.3 | 0.3 |

Table S5. Reference dose (RD) distributions reported in terms of median values for each figure of merit for both tuning and validation cohorts. No significant differences (p<0.05) were observed between cohorts.


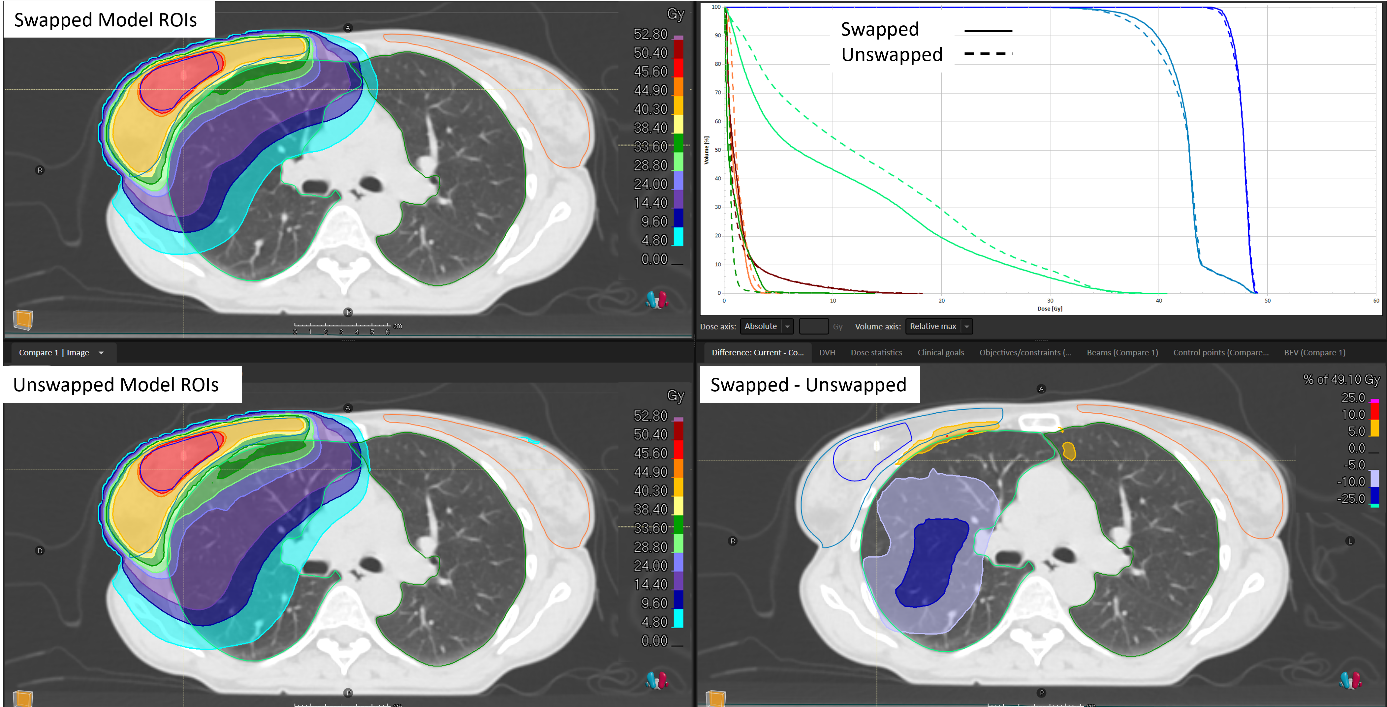


| **ROI** | **Technique** | **D98 [Gy]** | **Average [Gy]** | **D2 [Gy]** |
| --- | --- | --- | --- | --- |
| PTV_Boost | Swapped | 45.9 | 47.7 | 48.8 |
|  | Unswapped | 45.6 | 47.7 | 48.9 |
| PTV_Breast | Swapped | 36.4 | 42.4 | 48.0 |
|  | Unswapped | 35.4 | 42.2 | 48.0 |
| Breast_L | Swapped | NA | 1.0 | 2.7 |
|  | Unswapped | NA | 1.3 | 3.3 |
| Heart | Swapped | NA | 1.5 | 9.5 |
|  | Unswapped | NA | 1.3 | 9.9 |
| Lung_L | Swapped | NA | 0.9 | 3.7 |
|  | Unswapped | NA | 0.6 | 1.5 |
| Lung_R | Swapped | NA | 10.7 | 33.5 |
|  | Unswapped | NA | 13.5 | 34.0 |

Figure S2. Comparison between predicted doses (PD) in the axial plan for the Model ROIs swapping technique presented in this paper and the original unswapped Model ROIs association for a tuning cases. The DVH comparison reports cumulative curves for the PTV_Boost (dark blue), the PTV_Breast (light blue), the right lung (light green), the left lung (dark green), the heart (brown), and the left breast (orange). The table below reports the predicted dose statistics comparison, highlighting the large differences in the near maximum dose and average dose for left lung and right lung, respectively.


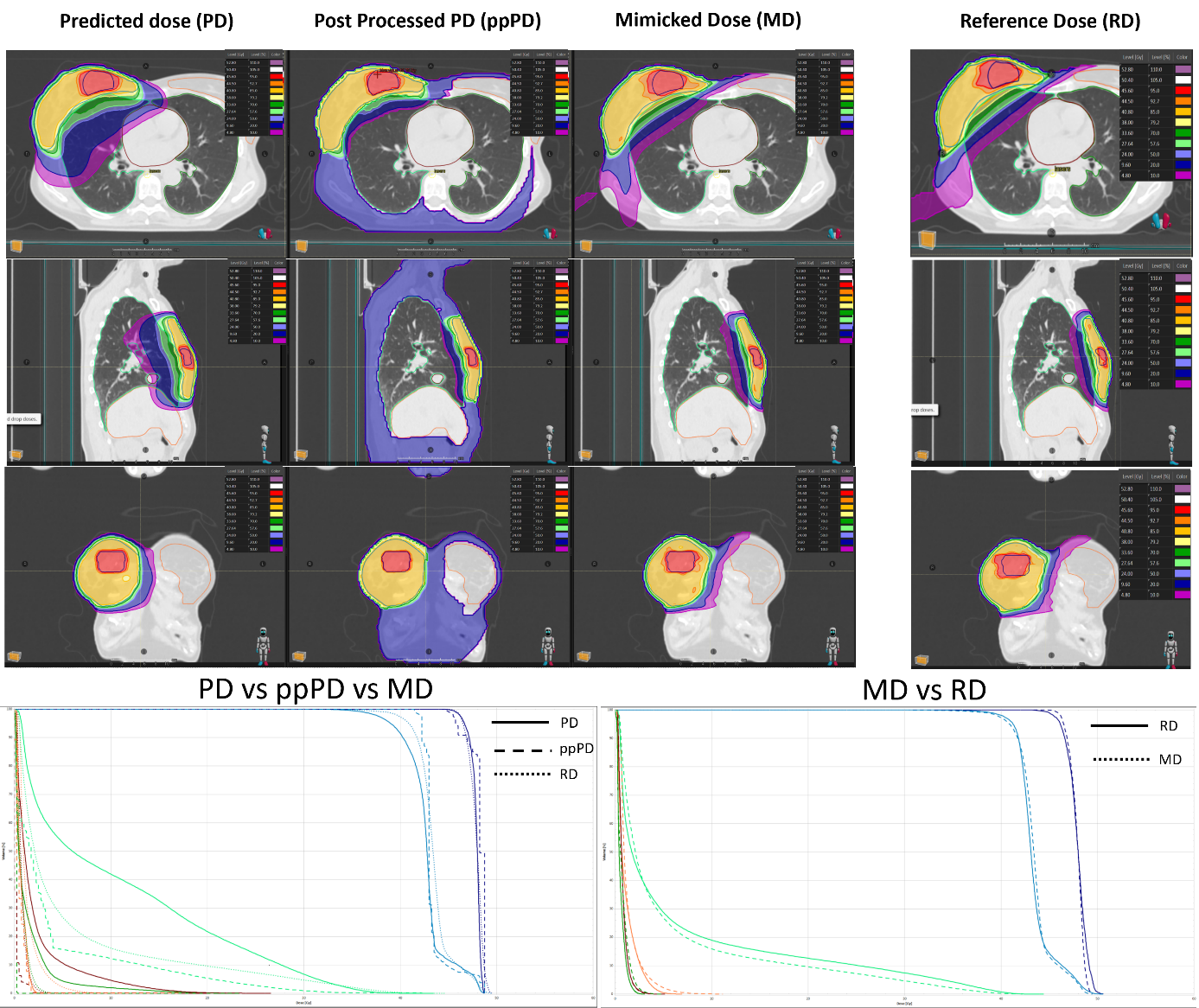


Figure S3. Sequential comparison between predicted dose (PD), post-processed predicted dose (ppPD) and mimicked dose (MD) against the manual reference dose (RD) in the three orthogonal planes (from the top to the bottom: transversal, sagittal, and coronal). The lower panel also shows the corresponding DVH comparison for the PTV_Boost (dark blue), the PTV_Breast (light blue), the right lung (light green), the left lung (dark green), the heart (brown), and the left breast (orange).
